# Practices, Attitudes, Perceived Knowledge, and Intentions Underlying Low‐Fat Dietary Behaviours in Adults Living With Bile Acid Diarrhoea: A Cross‐Sectional Study

**DOI:** 10.1111/jhn.70216

**Published:** 2026-02-11

**Authors:** Yvonne A. McKenzie, Christopher Todd, Calvin Heal, Sorrel Burden

**Affiliations:** ^1^ School of Health Sciences, Faculty of Biology, Medicine and Health University of Manchester Manchester UK; ^2^ Nuffield Health The Manor Hospital Oxford UK; ^3^ Manchester Academic Health Science Centre Manchester UK; ^4^ National Institute for Health and Care Research, Applied Research Collaboration Greater Manchester University of Manchester Manchester UK; ^5^ Manchester University NHS Foundation Trust Manchester UK; ^6^ Centre for Biostatistics, Faculty of Biology, Medicine, and Health, Manchester Academic Health Science Centre University of Manchester Manchester UK; ^7^ Salford Care Organisation, Northern Care Alliance NHS Trust Salford UK

**Keywords:** bile acid diarrhoea, cross‐sectional study, dietary behaviour, gastrointestinal symptoms, low‐fat diet, theory of planned behaviour

## Abstract

**Introduction:**

The effectiveness of low‐fats diet for managing bile acid diarrhoea (BAD) is unclear, yet many individuals may restrict fat intake. This study aims to explore factors associated with reducing fat intake.

**Methods:**

Cross‐sectional survey using convenience sampling recruited adults from the United Kingdom with a self‐reported diagnosis of BAD via online platforms (April to May 2021). Demographics, clinical characteristics, and low‐fat diet follower status data were collected. Potential predictors of intention to reduce fat intake were assessed using a modified validated questionnaire framed on the Theory of Planned Behaviour. Multinomial logistic regression was performed.

**Results:**

Of 434 respondents, current, past and non‐followers of low‐fat diets were 49%, 34% and 17%, whilst 79%, 83% and 78% reported chronic diarrhoea, respectively. Intention to reduce fat intake was associated with higher odds for six out of 20 predictor variables: current versus past‐followers, for its necessity for health (odds ratio [OR] = 1.4, 95% confidence interval [CI] 1.2 to 1.7), improving diarrhoea (OR = 1.5, 95% CI 1.3 to 1.7), abdominal pain (OR = 1.4, 95% CI: 1.3 to 1.7), bloating (OR = 1.4, 95% CI 1.2 to 1.6), flatulence (OR = 1.4, 95% CI 1.4 to 1.7), controlling gut symptoms (OR = 1.3, 95% CI 1.2 to 1.5); current versus non‐followers, for its necessary for health (OR = 1.3, 95% CI 1.1 to 1.5).

**Conclusion:**

Addressing the association between fat intake and an individual's attitude about its necessity for health and beliefs about controlling diarrhoeal symptoms may improve BAD management. For developing new therapies for symptom and body weight management, dietary behaviour components additional to fat intake warrant investigation.

## Summary

- This study found that many people living with bile acid diarrhoea continue to have diarrhoea despite treatment by bile acid sequestrants and low‐fat diets.
- Important predictors of intention to reduce fat intake were its necessity for health and improving diarrhoea, abdominal pain, bloating, flatulence, and controlling gut symptoms.
- In developing novel diet therapies for bile acid diarrhoea, aside from dietary fat intake reduction, other dietary modifications should be explored. Dietary modification to improve symptoms may differ from that to manage body weight.

## 1 Introduction

Low‐fat diets are used amongst people living with bile acid diarrhoea (BAD) to control symptoms [1]. At least 1 in every 100 adults have this debilitating gastrointestinal (GI) disorder [2], a rate that is rising [3]. Symptoms are characterised by urgent stool looseness, raised bowel frequency, abdominal pain, bloating, and flatulence [4]. In the clinical management of BAD, reducing fat intake is a next‐step therapeutic option following pharmacological treatment failure recommended by clinicians [3, 5, 6]. The hepatic production of bile acids and their secretion into the duodenum are a response to the ingestion of dietary fat. Bile acids are essential for the digestion and absorption of long‐chain fats and fat‐soluble vitamins A, D, E, and K [7, 8, 9]. When minimal dietary fat is consumed, the secretion of bile acids is minimised; the extent of watery stools and frequency of bowel movements can vary according to this [10]. However, within expert clinical practice guidelines, the evidence on the effectiveness of reducing fat intake was not synthesised [11]. A systematic review of diet intervention studies in BAD provided very low certainty of evidence on the effectiveness of reducing fat intake for improving diarrhoea, abdominal pain, and flatulence [12]. A recent survey of over 400 respondents from the United Kingdom (UK) with self‐reported BAD confirmed by ^75^selenium homocholic acid taurine (SeHCAT) testing found that 88% perceived they had food intolerances [13]. Fatty foods and dairy were frequently avoided, reported by 81% and 54%, respectively. From 39 foods, perceived triggers included takeaways, fish and chips, creamy sauces, and cream. However, diarrhoeal symptom provocation within 30 min of ingestion was evident for almost all listed food items, indicating importance of the gastro‐colonic reflex response.

There may be other influential factors for following a low‐fat diet in BAD that have not yet been explored. BAD is associated with gallstones, liver disease, and obesity [14, 15, 16]. This indicates a necessary clinical role for dietitians to support safe and sustainable reductions in body weight. Indeed, concern over unintentional weight gain has been raised by people living with BAD [1].

In understanding the benefit of following a low‐fat diet, a major barrier is that there is no consensus on what a ‘low‐fat diet’ is in BAD. Intervention studies in secondary BAD have quantified fat intake as a single target per day to assess effectiveness on GI symptoms. In one study published in 1979, 40 g of fat per day was used and food choices and preparation methods were given [17]. However, considering today's wide food choice in supermarkets, replication of this diet for longer‐term use is unlikely to be realistic. In two studies, the fat intake was estimated as 20% of total energy intake or expenditure, with no descriptions given of what to eat and drink [18, 19]. The definition of a ‘low‐fat diet’ is hindered because measuring fat‐related dietary behaviours is difficult [20], complicated by daily decision‐making on food choice and eating behaviours. Food choice has been defined as behaviours and other factors preceding food ingestion, influenced by preferences, food cost, purchasing frequency, which food product is purchased, food preparation, and intentions of what an individual plans to choose, buy, or consume [21]. Gaining knowledge on these factors is essential to ensure that people are adequately and appropriately treated and supported for long‐term self‐management of BAD.

The aim of this study is to explore factors associated with reducing dietary fat intake in BAD. The findings will contribute to the development of an evidence‐based dietary intervention for the clinical management of BAD following the Medical Research Council's guidance for developing and evaluating complex interventions [22].

## 2 Materials & Methods

### 2.1 Study Design

The study design was cross‐sectional using an online survey. This method provides a standardised and cost‐effective approach to collect quantitative data from respondents across the UK that is not limited by geography to determine prevalence and identify associations that may be useful for developing hypotheses [23]. Ethical approval was granted by The University of Manchester Research Ethics Committee (Review reference: 2021‐11232‐18456). Reporting follows the Strengthening of Observational Studies in Epidemiology guidelines and checklist for cross‐sectional studies [24] (Table Supplementary S1).

### 2.2 Participants

Participants self‐reported that they were adults ≥ 16 years of age with UK residency and a confirmed diagnosis of BAD by a gastroenterologist with SeHCAT testing to a 20% retention fraction cut‐off value in accordance with BAD severity classification [25]. Recruitment was by advertising of the survey on relevant online public UK domains: the BAD UK website, two Facebook groups: BAD UK and Bile Acid Malabsorption Support Group UK, the NHS Patient webinars website, and social media: X (formally Twitter). We collected anonymised data using an online questionnaire built on Research Electronic Data Capture (REDCap) [26, 27], designed to be completed in 20 min. However, this survey also collected self‐report data on food intolerance related to gastrointestinal symptoms, the results of which are published elsewhere [13]. Minimising of non‐response‐bias was addressed by having anonymity and considered important due to the sensitive nature of asking about bowel habits and medical history.

### 2.3 Questionnaire

**Figure 1:**
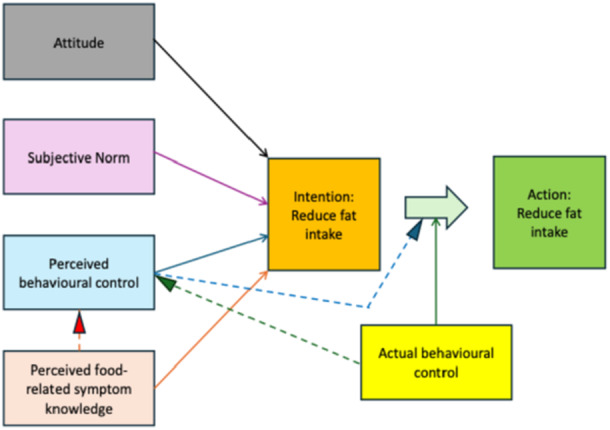
Theoretical framework of the theory of planned behaviour and perceived food‐related symptom knowledge to predict intention to reduce fat intake and perform the behaviour.

The questionnaire was developed by this research team following a review of the published literature on attitudes and behaviours related to reducing dietary fat intake. We adapted a validated 6‐item behaviour intervention questionnaire by Yardley et al. [28]. This is based on the widely used Theory of Planned Behaviour (TPB), which specifies precisely how the attitudes and intentions that predict behaviour should be measured [29, 30]. According to TPB, a behaviour is influenced by three main determinants of intention to perform the behaviour [29] (1) Attitude towards the behaviour, a favourable or unfavourable evaluation of the behaviour; (2) Subjective norm, a perceived social pressure to perform or not perform the behaviour; (3) Perceived behavioural control (PBC), a perception of capability, or perceived ease or difficulty, to perform the behaviour. Behavioural intention is the mediator between attitudes, subjective norm and PBC, the strongest predictor of reasoned behaviour. The intention to act is also influenced by how high the level of control actually is over the behaviour [29]. A predictor variable within the PBC construct was added which we termed ‘perceived food‐related symptom knowledge’ (PFSK). This was defined as the extent an individual believes that food improves their symptoms. The adapted model is shown in Figure 1.

Next, food choice factors from a dietary behaviour taxonomy [21] were applied to develop 20 statements. Six related to attitude, two to subjective norm, five to each of PBC and PFSK, and two to intention to reduce fat intake. The definitions of these constructs to perform the behaviour of reducing fat are shown in Table S2 which presents the questionnaire. For PFSK, the gastrointestinal symptoms selected were those used in the first part of the survey. One further predictor variable was included to measure symptoms globally. The scores for the 7‐point scale ranged from strongly disagree to strongly agree. To reduce responder burden, two further response options were included: ‘I don't know’ and ‘Not applicable’.

At the beginning of the questionnaire, five items were included to understand respondents' dietary practices in general and then specifically to following a low‐fat diet or not (shown in Table S2). Low‐fat diet follower status was self‐reported, defined as a current, past, or non‐follower of a low‐fat diet. A non‐follower was a respondent who identified as having never tried to follow a low‐fat diet.

### 2.4 Data Items and Measurement

All collected data items were self‐reported and included SeHCAT severity, length of time in years and months since BAD diagnosis, BAD medication usage, and baseline characteristics as listed in Table 1. The effectiveness of BAD medication to treat diarrhoeal symptoms was measured via a visual analogue scale (0–100). Stool consistencies were measured using the Bristol stool form scale [31], with diarrhoea defined as type 6 or 7 that was ongoing in the last 4 weeks. To support accuracy of height and body weight, conversion charts of imperial and metric measurement values were provided.

**Table 1:** Demographic and clinical characteristics of respondents.

	Whole sample *N* = 434	Low‐fat diet follower group		
		Current‐followers *n* = 211	Past‐followers *n* = 149	Non‐followers *n* = 74
Female, *n* (%)	358 (82.5)	176 (83.4)	132 (88.6)	50 (67.6)
Male, *n* (%)	57 (13.1)	19 (9.0)	15 (10.1)	23 (31.1)
Other/Preferred not to say, *n* (%)	19 (4.4)	16 (7.6)	2 (1.3)	1 (1.4)
Median age, years (IQR) *n* = 433 (1 missing)	49.0 (38.0–60.0)	50.5 (39.0–62.0)	47.0 (36.5–56.5)	49.8 (38.5–60.0)
Median BMI, kg/m^2^ (IQR) *n* = 423 (11 missing)	28.7 (24.4–33.8)	28.4 (24.4–33.7)	31.8 (27.8–37.7)	27.2 (23.4–30.2)
Ethnic group, *n* (%)				
White	409 (94.2)	191 (90.5)	147 (98.7)	71 (96.0)
Non‐White/preferred not to say	25 (5.8)	20 (9.5)	2 (1.3)	3 (4.1)
Highest level of educational qualification achieved, *n* (%)				
None	10 (2.3)	7 (3.3)	3 (2.0)	3 (4.1)
Trade/NVQ	51 (11.8)	17 (8.1)	21 (14.1)	20 (27.0)
GCSE or equivalent	86 (19.8)	49 (23.2)	25 (16.8)	12 (16.2)
Diploma	97 (22.4)	53 (25.1)	32 (21.5)	12 (16.2)
University degree(s)	164 (37.8)	65 (30.8)	65 (43.6)	34 (46.0)
Preferred not to say	26 (6.0)	20 (9.5)	3 (2.0)	3 (4.1)
Main occupation, *n* (%)				
Working, full‐time	165 (38.0)	66 (31.3)	71 (47.7)	28 (37.8)
Working, part‐time	82 (18.9)	42 (19.9)	26 (17.5)	14 (18.9)
Retired	76 (17.5)	45 (21.3)	17 (11.4)	14 (18.9)
Unable to work	63 (14.5)	35 (16.6)	19 (12.8)	9 (12.2)
Homemaker or other^a^	48 (11.1)	23 (10.9)	16 (10.7)	9 (12.2)
Severity of BAD defined by SeHCAT^(26)^, *n* (%)				
Severe (< 5%)	199 (45.9)	104 (49.3)	61 (40.9)	34 (46.0)
Moderate (5 to < 10%)	110 (25.3)	42 (19.9)	48 (32.2)	20 (27.0)
Mild (10 to < 15%)	40 (9.2)	22 (10.4)	14 (9.4)	4 (5.4)
Borderline (> 15%–20%)/Did not know	85 (19.6)	43 (20.4)	26 (17.5)	16 (21.6)
Persisting diarrhea *n* (%) *n* = 416 (18 missing)	333 (80.0)	155 (78.7)	123 (83.1)	55 (77.5)
Median duration of BAD, years (IQR)	3.0 (1.0–5.0)	3.0 (1.0–5.0)	2.0 (1.0–4.0)	4.0 (2.0–6.0)
Other current medical conditions or past treatments, *n* (%)				
None	35 (8.1)	18 (8.5)	6 (4.0)	11 (14.9)
IBD	55 (12.7)	27 (12.8)	18 (12.1)	10 (13.5)
IBS	161 (37.1)	71 (33.6)	70 (47.0)	20 (27.0)
Cholecystectomy	180 (41.5)	91 (43.1)	70 (47.0)	19 (25.7)
Other GI surgery	43 (9.9)	18 (8.5)	13 (8.7)	12 (16.2)
Cancer	31 (7.1)	19 (9.0)	6 (4.0)	6 (8.1)
Coeliac disease	19 (4.4)	10 (4.7)	5 (3.4)	4 (5.4)
Type 2 diabetes	33 (7.6)	19 (9.0)	10 (6.7)	4 (5.4)
Fatty liver	68 (15.7)	33 (15.6)	26 (17.5)	9 (12.2)
Hyperlipidaemia	22 (5.1)	7 (3.3)	11 (7.4)	4 (5.4)
Anxiety	173 (39.9)	72 (34.1)	72 (48.3)	29 (39.2)
Depression	143 (33.0)	64 (30.3)	51 (34.2)	28 (37.8)
Lactose intolerance	35 (8.1)	19 (9.0)	12 (8.1)	4 (5.4)
Other	117 (27.0)	59 (28.0)	44 (29.5)	14 (18.9)
BAD medication, *n* (%)				
Colestyramine (1–6 sachets per day)	107 (24.7)	47 (22.3)	37 (24.8)	23 (31.1)
Colestipol (1–4 sachets per day)	11 (2.5)	5 (2.4)	2 (1.3)	4 (5.4)
Colesevelam (number of tablets taken per day)				
1–4	167 (38.5)	85 (40.3)	57 (38.3)	25 (33.8)
5–6	98 (22.6)	50 (23.7)	36 (24.2)	12 (16.2)
Loperamide	214 (49.3)	101 (47.9)	76 (51.0)	37 (50.0)
None of these medicines	31 (7.1)	16 (7.6)	11 (7.4)	4 (5.4)
Median effectiveness of medication (%) (IQR)	65 (36.5 to 78.0)	61.0 (31.0, 80.0)	66.0 (38.0, 75.0)	68.0 (50.0, 80.0)

Abbreviations: BAD, bile acid diarrhoea; BMI, body mass index; CI, confidence interval; GCSE, General Certificate of Secondary Education; GI, gastrointestinal; IBD, Inflammatory bowel disease; IBS, Irritable bowel syndrome; IRQ, interquartile range; NVQ, National Vocational Qualification; SeHCAT: ^75^selenium homocholic acid taurine.

^a^ Includes: full‐time student; looking for work; out of work and not looking for work; shift worker; other; preferred not to say.

As this research was exploratory, no formal validation of the questionnaire was undertaken. A target sample size was not estimated, although this was calculated post hoc. Based on a UK's population prevalence rate of BAD of 1% (approximately 533,700 people at the time), to achieve a 95% confidence interval (CI) with a 5% margin of error, a minimum sample size of 384 would be required [32]. Therefore, a target of 400 respondents provides sufficient accuracy in the estimates. The survey was pretested on mobile phones and computers for content clarity by 12 experts in BAD and nutrition (patient representatives, lay people, healthcare professionals, and academics).

### 2.5 Analysis

Statistical analyses were performed using SPSS version 28 (IBM, USA). Summary statistics of continuous variables are presented as mean with standard deviation or median and interquartile range depending on normality. Categorical variables are reported as counts (n) and percentages (%) with 95% CI [33]. The Kruskal‐Wallis H test [34] was used with the pairwise comparisons adjusted by the Bonferroni post‐hoc correction for multiple tests to determine whether and where any between‐group differences in agreement score (0 to 6) for each predictor (independent) variable lie. Responses for the scores, ‘I don't know’ and ‘Not applicable’ were handled as ‘missing’ items and removed from the denominator. The reduced counts are presented in Table S2. We then performed multinomial logistic regression to estimate the magnitude of the likelihood of intention to reduce fat intake between the three groups for each of the 20 constructs, having checked that the assumptions of the analysis were met. Each construct was entered into its own multinomial logistic regression model, with ‘intention to reduce fat’ group as the outcome, alongside a common, pre‐specified list of potential confounders: age and body mass index (BMI), education status (binary variable: higher or lower level) and work status (binary variable: at home or away from home). Neither biological sex nor ethnicity grouping are used because of the small numbers of males and ethnic minority members in the sample. Outcomes are presented as odds ratio (OR) along with 95% CI and *p*‐value. *p*‐values are adjusted using the Holm‐Bonferroni sequential correction for multiple tests [35]. The significance level is defined as *α* = 0.05.

## 3 Results

The online survey was conducted from 29 April to 27 May 2021.

### 3.1 Respondents' Characteristics

Of 434 respondents who completed the online questionnaire, 48.6% reported following a low‐fat diet (current‐followers). Past‐followers and those who had never tried following a low‐fat diet (non‐followers) comprised of 34.3% and 17.0% of the sample, respectively. Respondents' demographic and clinical characteristics are summarised in Table 1. First and second‐line BAD medication was used by 92.6% of all respondents. In current, past, and non‐followers, chronic diarrhoea was reported by 78.7% (155/197, 95% CI 72.3 to 84.2), 83.1%, (123/148, 95% CI 76.1 to 88.8), and 77.5%, (55/71, 95% CI 66.0 to 86.5), respectively. The prevalence of reported gallbladder removal was lower in non‐followers compared to past‐followers. No other between‐group differences in the prevalence rates of the respondents' characteristics were found.

### 3.2 Do People Living With BAD Have Enough Time to Look After Their Own Diet?

Of the whole sample, 64.3% reported they had adequate time to look after their own diet. A higher proportion of current‐followers had adequate time to look after their own diet compared to past‐followers: 74.4% (157/211, 95% CI 68.0 to 80.2) versus 52.4% (78/149, 95% CI 44.0 to 60.6). For non‐followers, the proportion was 59.5% (44/74, 95% CI 47.4 to 70.7).

The proportions with reportedly inadequate time to look after their own diet in current, and non‐followers were 15.6% (33/211, 95% CI 11.0 to 21.3), 32.9% (49/149, 95% CI 25.4 to 41.1) and 27.0% (20/74, 95% CI 17.4 to 38.6), respectively.

### 3.3 Duration of Following a Low‐Fat Diet

How long respondents reported adhering to low‐fat diets amongst current and past‐followers is shown in Table 2. Amongst current‐followers, 74.4% (157/211, 95% CI 68.3, 80.5) reported long‐term adherence (over 12 months). Amongst past‐followers, 55.7% (83/149, 95% CI 47.4, 63.8) had given up within 6 months.

**Table 2:** Duration of following a low‐fat diet amongst current and past‐followers (*n*= 359).

Duration	Current‐followers		Past‐followers	
	*n* = 210 (%) (1 missing)	95% CI	*n* = 149 (%)	95% CI
Longer than 5 years	76 (36.2)	29.7–43.1	8 (5.4)	2.4–10.3
Over 1 year	81 (38.6)	32.0–45.5	32 (21.5)	15.2–29.0
Less than 12 months	9 (4.3)	2.0–8.0	26 (17.5)	11.7–24.5
Less than 6 months	19 (9.1)	5.5–13.8	23 (15.4)	10.0–22.3
Less than 3 months	19 (9.1)	5.5–13.8	39 (26.2)	19.3–34.0
Less than 4 weeks	6 (2.9)	1.1–6.1	21 (14.1)	8.9–20.7

Abbreviation: CI, confidence interval.

### 3.4 Reason for Following a Low‐Fat Diet

A higher proportion of current than past‐followers were following low‐fat diets to manage BAD, the most cited of the four reasons for following the dietary restriction, See Table 3. For managing body weight, a higher proportion of past than current‐followers followed low‐fat diets. For managing their health or another reason, smaller and similar proportions of each group followed low‐fat diets.

**Table 3:** Reason for following a low‐fat diet amongst current and past followers (*n* = 360).

Reason for following a low‐fat diet	Current‐followers (*n* = 211)		Past‐followers (*n* = 149)	
	(%)	95% CI	(%)	95% CI
To help me to manage my BAD	147 (69.7)	63.0 to 75.8	61 (40.9)	33.0 to 49.3
To help me to manage my weight	26 (12.3)	8.2 to 17.5	66 (44.3)	36.2 to 52.7
To help me to look after my health	17 (8.1)	4.8 to 12.6	9 (6.0)	2.8 to 11.2
Another reason	21 (10.0)	6.3 to 14.8	13 (8.7)	4.7 to 14.5

Abbreviation: CI, confidence interval.

### 3.5 Useful Sources of Information

**Figure 2:**
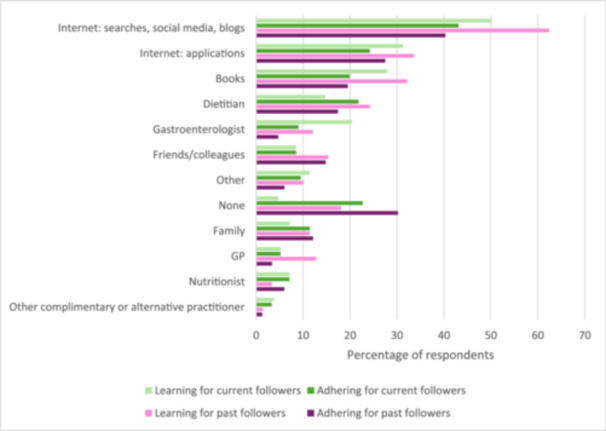
Useful sources of information for learning about and adhering to a low‐fat diet in current and past followers (*n* = 360).

The most popular of the twelve useful sources of information for learning about and adhering to a low‐fat diet was the Internet, followed by dietitians and books (Figure 2). For learning about low‐fat diets, a higher proportion of current than past‐followers found that dietitians were useful (30.8%, 65/211, 95% CI 24.7 to 37.5 compared to 20.8%, 31/149, 95% CI 14.6 to 28.2). For all of the other information sources for learning about and adhering to low‐fat diets, the proportions of current and past‐followers were similar.

### 3.6 Factors Associated With Reducing Fat Intake

Outcomes on agreement scores for reducing fat intake by low‐fat diet follower status are summarised in Table S3.

Outcomes of the multinomial logistic regression identified 14 associations between six predictor variables and the three low‐fat diet follower categories, highlighted in Table 4.

**Table 4:** Multinomial logistic regression for the association of predictive factors with intention to reduce fat intake adjusted for age, body mass index, level of education and work status.

Predictor variable	Reference category: non‐follower		Reference category: past‐follower		Reference category: current‐follower	
	Adjusted odds ratio (95% CI)	*p* value	Adjusted odds ratio (95% CI)	*p* value	Adjusted odds ratio (95% CI)	*p* value
**Attitude:** Having less fat in my diet …						
Could be beneficial to my health	C: 1.27 (1.06–1.52)	0.33	C: 1.25 (1.06–1.47)	0.24	P: 0.80 (0.68–0.94)	0.22
	P: 1.02 (0.85–1.22)	1.00	N: 0.98 (0.82–1.18)	1.00	N: 0.79 (0.66–0.94)	0.26
Is necessary for my health	C: 1.43 (1.20–1.71)	**0.04**	C: 1.31 (1.12–1.53)	**0.04**	P: 0.76 (0.65–0.89)	**0.04**
	P: 1.09 (0.92–1.30)	1.00	N: 0.92 (0.77–1.09)	1.00	N: 0.70 (0.59–0.83)	**0.04**
Would be pleasant for me	C: 1.31 (1.10–1.55)	0.08	C: 1.21 (1.05–1.38)	0.26	P: 0.83 (0.72–0.95)	0.24
	P: 1.09 (0.91–1.30)	1.00	N: 0.92 (0.77–1.10)	1.00	N: 0.77 (0.65–0.91)	0.07
Would be convenient for me	C: 1.19 (1.00–1.40)	1.00	C: 1.18 (1.03–1.36)	0.61	P: 0.85 (0.74–0.97)	0.52
	P: 1.00 (0.84–1.21)	1.00	N: 1.0 (0.83–1.20)	1.00	N: 0.84 (0.71–1.00)	1.00
Food costs would be less	C: 1.01 (0.83–1.21)	1.00	C: 1.01 (0.87–1.17)	1.00	P: 0.99 (0.85–1.15)	1.00
	P: 1.00 (0.81–1.22)	1.00	N: 1.01 (0.82–1.23)	1.00	N: 1.00 (0.83–1.20)	1.00
Groceries: I would not need extra time	C: 0.95 (0.81–1.12)	1.00	C: 0.92 (0.80–1.05)	1.00	P: 1.09 (0.95–1.24)	1.00
	P: 1.04 (0.87–1.24)	1.00	N: 0.97 (0.81–1.15)	1.00	N: 1.05 (0.89–1.24)	1.00
Food prep: I would not need extra time	C: 0.95 (0.81–1.11)	1.00	C: 0.94 (0.82–1.07)	1.00	P: 1.07 (0.94–1.22)	1.00
	P: 1.01 (0.85–1.21)	1.00	N: 0.99 (0.83–1.17)	1.00	N: 1.06 (0.90–1.24)	1.00
**Subjective norm:** People important to me, such as: i. my family, friends and doctor, think that I should eat less fatty food; ii. my family and friends, do not have lots of fatty or fried food in their usual diet						
i.	C: 1.03 (0.88–1.21)	1.00	C: 1.00 (0.88–1.14)	1.00	P: 1.00 (0.88–1.14)	1.00
	P: 1.03 (0.87–1.22)	1.00	N: 0.97 (0.82–1.15)	1.00	N: 0.97 (0.83–1.13)	1.00
ii.	C: 1.14 (0.96–1.34)	1.00	C: 1.13 (0.99–1.29)	1.00	P: 0.89 (0.78–1.02)	1.00
	P: 1.01 (0.85–1.20)	1.00	N: 0.99 (0.83–1.18)	1.00	N: 0.88 (0.75–1.04)	1.00
**PBC:** If I wanted to, it/I would … to have less fat in my diet						
Be easy	C: 0.99 (0.84–1.16)	1.00	C: 1.01 (0.88–1.15)	1.00	P: 0.99 (0.87–1.13)	1.00
	P: 0.98 (0.82–1.17)	1.00	N: 1.02 (0.86–1.22)	1.00	N: 1.01 (0.86–1.19)	1.00
Know what I would have to do	C: 1.26 (1.07–1.48)	0.27	C: 1.18 (1.03–1.36)	0.62	P: 0.85 (0.73–0.97)	0.52
	P: 1.06 (0.90–1.26)	1.00	N: 0.94 (0.79–1.12)	1.00	N: 0.80 (0.68–0.94)	0.22
Have the self‐discipline	C: 1.10 (0.94–1.30)	1.00	C: 1.18 (1.04–1.35)	1.00	P: 0.85 (0.74–0.96)	0.32
	P: 0.93 (0.78–1.10)	1.00	N: 1.08 (0.91–1.28)	1.00	N: 0.91 (0.77–1.07)	1.00
Be completely up to me…	C: 0.97 (0.83–1.13)	1.00	C: 1.07 (0.95–1.21)	1.00	P: 0.93 (0.82–1.05)	1.00
	P: 0.90 (0.77–1.06)	1.00	N: 1.11 (0.94–1.30)	1.00	N: 1.03 (0.88–1.21)	1.00
**Perceived food‐related symptom knowledge:** Having less fat in my diet might …						
Improve my diarrhoea	C: 1.25 (1.05–1.49)	0.47	C: 1.47 (1.27–1.69)	**0.04**	P: 0.68 (0.59–0.79)	**0.04**
	P: 0.85 (0.71–1.01)	1.00	N: 1.18 (0.99–1.40)	1.00	N: 0.80 (0.67–0.96)	0.28
Improve my abdominal pain	C: 1.25 (1.04–1.49)	0.70	C: 1.42 (1.23–1.65)	**0.04**	P: 0.70 (0.60–0.82)	0.04
	P: 0.88 (0.73–1.05)	1.00	N: 1.14 (0.95–1.38)	1.00	N: 0.80 (0.67–0.96)	0.52
Improve my bloating	C: 1.15 (0.97–1.37)	1.00	C: 1.36 (1.18–1.58)	**0.04**	P: 0.74 (0.64–0.85)	**0.04**
	P: 0.85 (0.70–1.02)	1.00	N: 1.18 (0.98–1.43)	1.00	N: 0.87 (0.73–1.04)	1.00
Improve my wind	C: 1.19 (0.99–1.42)	1.00	C: 1.36 (1.17–1.59)	**0.04**	P: 0.74 (0.63–0.86)	**0.04**
	P: 0.87 (0.72–1.06)	1.00	N: 1.15(0.95–1.39)	1.00	N: 0.84 (0.70–1.01)	1.00
Help me to control my gut symptoms	C: 1.17 (0.99–1.38)	1.00	C: 1.33 (1.15–1.52)	**0.04**	P: 0.75 (0.66–0.87)	**0.04**
	P: 0.89 (0.74–1.05)	1.00	N: 1.13 (0.95–1.34)	1.00	N: 0.85 (0.72–1.01)	1.00
**Behavioural intention:** If offered the guidance/opportunity, I intend to cut back on how much fat is in my diet.						
Guidance	C: 1.08 (0.92–1.28)	1.00	C: 1.09 (0.95–1.25)	1.00	P: 0.92 (0.80–1.06)	1.00
	P: 1.00 (0.84–1.19)	1.00	N:1.00 (0.84–1.20)	1.00	N: 0.92 (0.78–1.09)	1.00
Opportunity	C: 1.05 (0.90–1.23)	1.00	C: 1.04 (0.91–1.19)	1.00	P: 0.96 (0.84–1.10)	1.00
	P: 1.01 (0.85–1.19)	1.00	N: 0.99 (0.84–1.18)	1.00	N: 0.95 (0.82–1.12)	1.00

Abbreviations: C, current followers; CI, confidence interval; Food prep, food preparation; N, non‐followers; P‐past followers.

### 3.7 Attitude

Current‐followers when compared to non‐followers and past‐followers, were 1.43 times and 1.31 times more likely than not to believe that reducing fat intake is necessary for their health (95% CI 1.20 to 1.71; *p* = 0.04% and 95% CI 1.12 to 1.53; *p* = 0.04), respectively. When non‐followers were compared to past‐followers, no association was found.

No associations were found for the attitude constructs on benefit to health, pleasantness, convenience, cost or time for grocery shopping and food preparation. This indicates that these factors were not associated with reducing fat intake in BAD.

### 3.8 Subjective Norm, Perceived Behavioural Control, and Intention

For all group comparisons, none of the constructs of subjective norm, PBC, and behavioural intention, increased or decreased the likelihood of reducing fat intake.

### 3.9 Perceived Food‐Related Symptom Knowledge

Current‐followers when compared to past‐followers were more likely than not to believe that reducing fat intake improves their diarrhoea (OR = 1.47, 95% CI 1.27 to 1.69; *p* = 0.04), abdominal pain (OR = 1.42, 95% CI 1.23 to 1.65; *p* = 0.04), bloating (OR = 1.36, 95% CI 1.18 to 1.58; *p* = 0.04), flatulence (OR = 1.36, 95% CI 1.17 to 1.59; *p* = 0.04), and control of gut symptoms (OR = 1.33, 95% CI 1.15 to 1.52; *p* = 0.04). When non‐followers were compared to past‐followers, no associations were found.

## 4 Discussion

This study explored attitudes and behaviours associated with reducing dietary fat intake in adults living with BAD. There are five key findings. First, in this sample of 434 self‐reporting respondents, a high proportion (83%) were not naïve to low‐fat diets: half of the sample reported following low‐fat diets, of which three‐quarters had been doing so for over 12 months. Dietitians were found to be more useful to current than past‐followers for learning about low‐fat diets, however this difference diminished for adherence to the dietary restriction. According to the published literature [5], a registered dietitian should be able to offer greatest support out of all of the information sources listed in the survey. A recent UK survey amongst hospitals within all National Health Service trusts in England indicates an inequity of gastrointestinal dietetic services across England and limited consultation times for patients [36]. In our study, the most frequently cited source for information about learning and adhering to low‐fat diets was reportedly the Internet. Misinformation about nutrition and diet on social media networks is a known concern for public health institutions, patient associations and individuals [37, 38, 39]. Until the evidence‐base on diet therapies for BAD strengthens from its current very weak position through research, having reliable, comprehensive diet resources in BAD for both patients and healthcare professionals and access to the services of registered dietitians will remain challenging.

Secondly, it is perhaps surprising that 80% of respondents reported persisting diarrhoea, and this proportion was similar across all three low‐fat follower status groups. There is a paucity of evidence on the clinical course of BAD. The prevalence of diarrhoea in our study is similar to that identified in a Danish survey [5]. Participants were recruited from a hospital setting where BAD was diagnosed by abnormal SeHCAT scores to a 15% retention fraction cut‐off value. ‘Bothersome’ diarrhoea was self‐reported in 74% of 273 responders; 49% reported that since their diagnosis of BAD, diarrhoea was worse or unaltered. In a 12‐year longitudinal study from the UK in 58 clinician‐assessed patients with mean SeHCAT scores of 2.8%, 34% of patients had persisting diarrhoea due to intolerance to medication and no treatment. Of the 66% who were on medication, the proportion that did not have adequate relief from diarrhoea is unclear. Our finding contributes to the evidence showing that in the long‐term, many people with BAD continue to have persisting diarrhoea for which novel therapies need to be developed.

A potential explanation for ongoing diarrhoea is inadequate fat intake reduction due to underestimation of the level of fat intake tolerated. This may be on a meal by meal basis rather than per day since circulating intestinal fibroblast growth factor 19 modulates hepatic bile acid synthesis [40, 41]. Adherence to dietary fat restriction long‐term is known to be difficult [42, 43]. Evidence from a Dutch population study in 1507 adults comparing self‐rated fat intakes with objective assessments of fat intakes showed a high prevalence of underestimating fat intake [44], and men were more unrealistic than women. Improving dietary fat intake awareness by objectively assessing fat intake was recommended. In studies that have measured fat intake from participant‐completed prospective food records, nutritional awareness and building one's own nutritional knowledge base have been identified as important for adhering to low‐fat diets as a lifelong commitment [43, 45]. Research is needed to (1) identify what level of fat intake is effective for the diarrhoea of BAD, (2) develop behavioural interventions designed for lifelong adherence, detailing food portion sizes and meal pattern across a day for accurate quantification of fat intake. Authors from this group have proposed a dietary pattern with a fat intake of 8 g per meal across five smaller meals per day, embedded into lifestyle [46].

Third, intention to reduce fat intake was found to be associated with its necessity for health, and improving diarrhoea, abdominal pain, bloating, flatulence, and controlling gut symptoms when current‐followers were compared to past‐followers. The other 14 of 20 attitudinal, subjective norm, and PBC factors were not associated with this dietary behaviour. This indicates that for those following low‐fat diets, motivation to reduce fat intake is focused on both symptom control and health. Therefore, dietary change is a major concern of theirs within daily living. In other studies, health and behaviour/lifestyle modification were similarly important factors for adhering to dietary fat restriction [43, 47]. In this study, health was not defined and could have been interpreted to encompass mental as well as general health. Nonetheless, this study's findings on these six factors, its necessity for health, and improving diarrhoea, abdominal pain, bloating, flatulence, and controlling gut symptoms, are compatible with clinically important and meaningful patient‐reported outcomes in BAD, of improving health‐related quality of life and debilitating symptoms [1, 4, 48, 49].

Fourth, those who had previously followed low‐fat diets were more likely than not to believe that reducing fat intake was not necessary for their health and did not improve diarrhoea, abdominal pain, bloating, flatulence, or control gut symptoms. A higher proportion of past‐followers than current‐followers used low‐fat diets for managing body weight. Nearly half of past‐followers continued dietary fat restriction beyond 6 months. It is relevant to note that our study did not survey those who may have been using low‐fat diets to manage both BAD and body weight. Bile acids have a key role in the metabolism of lipids and glucose [50, 51]. Bile acid metabolic dysregulation in BAD is linked to many comorbidities including raised BMI [16, 52]. Different dietary intervention designs may be warranted for managing weight, the diarrhoea of BAD, or both. For past‐followers of low‐fat diets, their beliefs about diet and food need to be understood to develop new frameworks or theories of what might work for them for managing body weight, as well as any ongoing diarrhoea.

Fifth, the intention constructs showed no associations with intention to reduce fat intake by low‐fat diet follower status after adjusting for the potential confounders, age, BMI, level of education, and work status. The data suggest that reducing fat intake has a role in a proportion of people living with BAD. Research is needed to develop diet therapies that are more comprehensive than reducing fat intake, that offer a whole‐diet intervention, personalised in accordance with an individual's motivations and behaviours pertaining to their health, diarrhoeal symptoms, and body weight.

### 4.1 Strengths and Limitations of the Study

A strength of this study is its sample size. This suggests that further studies conducted remotely via participants accessing their own digital devices is appropriate for not limiting participation by geographical location. A further strength is a robust statistical approach which saw each independent variable of interest tested in its own model with a set of pre‐specified confounding variables. However, the data were observational in design and cannot be extrapolated to determine causality. Randomised controlled trials of dietary interventions that include accurate measurement of fat intake by participants would strengthen the evidence on the role of reducing fat intake for managing diarrhoea and symptoms in BAD.

The use of convenience sampling is a major limitation which restricts the generalisability of the findings to the wider population living with BAD. The sample is probably under‐representative of men and those of non‐white and lower educational backgrounds. Demographic data on the UK population living with BAD in the community are lacking. Thus speculation about quite how representative the current sample is may be unfounded. Nevertheless, selection bias may have occurred because (i) respondents are likely to have been interested in diet because the survey was on diet, and (ii) inadequate relief from diarrhoea using medication may also have motivated participation in the survey. Self‐report data are also a main limitation, causing unavoidable risk of recall bias and measurement bias due to the Hawthorne effect, respondent fatigue, and straight line answering. Our survey's anonymity and that responders could return to answering questions at their own pace may have limited these. Not providing a definition for ‘low‐fat diet’ left open its interpretation to respondents. However, it seems unlikely that participants would have allocated themselves to the incorrect group.

## 5 Conclusion

This study showed that important predictors of intention to reduce fat intake are its necessity for health and improving or controlling diarrhoeal symptoms. Using these constructs for designing novel dietary interventions may help people living with BAD to obtain greater success in managing this debilitating medical condition.

## Author Contributions

Yvonne McKenzie conceived the idea for the study. Yvonne McKenzie, Chris Todd, and Sorrel Burden contributed to study design. Yvonne McKenzie was responsible for developing the survey on REDCap, recruitment, and data collection. Yvonne McKenzie, Chris Todd, Calvin Heal, and Sorrel Burden contributed to data analysis and interpretation. Yvonne McKenzie drafted the manuscript. All authors provided critical feedback and contributed to the final version of the manuscript. All authors approved the final version of the manuscript, and it has not been published elsewhere.

## Funding

The authors received no specific funding for this work.

## Ethics Statement

The name of the ethics committee and approval number: The University of Manchester Research Ethics Committee (Review reference: 2021‐11232‐18456).

## Conflicts of Interest

The authors declare no conflicts of interest.

## Supporting information


**Table S1:** STROBE Statement Checklist. **Table S2:** Questionnaire. **Table S3:** Agreement scores for the predictor variables on intention to reduce fat intake.
