# Supplementary material for: Practices, Attitudes, Perceived Knowledge, and Intentions Underlying Low‐Fat Dietary Behaviours in Adults Living With Bile Acid Diarrhoea: A Cross‐Sectional Study
Source: J Hum Nutr Diet. 2026 Feb 11;39(1):e70216. doi: 10.1111/jhn.70216 (PMC12895096; doi:10.1111/jhn.70216)
Supplement: Supplementary file 1 — Table S1: STROBE Statement Checklist. Table S2: Questionnaire. Table S3: Agreement scores for the predictor variables on intention to reduce fat intake. [file JHN-39-0-s001.docx]

**SUPPORTING INFORMATION**

**Contents**

TABLE S1 STROBE Statement Checklist

TABLE S2 Questionnaire

TABLE S3 Agreement scores for the predictor variables on intention to reduce fat intake

TABLE S1 **STROBE Statement—Checklist**

|  | Item No | Recommendation | Page No |
| --- | --- | --- | --- |
| **Title and abstract** | 1 | (*a*) Indicate the study’s design with a commonly used term in the title or the abstract | 1 |
|  |  | (*b*) Provide in the abstract an informative and balanced summary of what was done and what was found | 2 |
| Introduction | | | |
| Background/rationale | 2 | Explain the scientific background and rationale for the investigation being reported | 4-5 |
| Objectives | 3 | State specific objectives, including any prespecified hypotheses | 5 |
| Methods | | | |
| Study design | 4 | Present key elements of study design early in the paper | 5 |
| Setting | 5 | Describe the setting, locations, and relevant dates, including periods of recruitment, exposure, follow-up, and data collection | 4, 7 |
| Participants | 6 | (*a*) Give the eligibility criteria, and the sources and methods of selection of participants | 4 |
| Variables | 7 | Clearly define all outcomes, exposures, predictors, potential confounders, and effect modifiers. Give diagnostic criteria, if applicable | 6 and SI, Table S2 |
| Data sources/ measurement | 8* | For each variable of interest, give sources of data and details of methods of assessment (measurement). Describe comparability of assessment methods if there is more than one group | 4, 6, Table 1 |
| Bias | 9 | Describe any efforts to address potential sources of bias | 4 |
| Study size | 10 | Explain how the study size was arrived at | 6 |
| Quantitative variables | 11 | Explain how quantitative variables were handled in the analyses. If applicable, describe which groupings were chosen and why | 6 |
| Statistical methods | 12 | (*a*) Describe all statistical methods, including those used to control for confounding | 7 |
|  |  | (*b*) Describe any methods used to examine subgroups and interactions | 7 |
|  |  | (*c*) Explain how missing data were addressed | 7 |
|  |  | (*d*) If applicable, describe analytical methods taking account of sampling strategy | N/A |
|  |  | (*e*) Describe any sensitivity analyses | N/A |
| Results | | | |
| Participants | 13* | (a) Report numbers of individuals at each stage of study—eg numbers potentially eligible, examined for eligibility, confirmed eligible, included in the study, completing follow-up, and analysed | 8 |
|  |  | (b) Give reasons for non-participation at each stage | N/A |
|  |  | (c) Consider use of a flow diagram | - |
| Descriptive data | 14* | (a) Give characteristics of study participants (eg demographic, clinical, social) and information on exposures and potential confounders | 8, Table 1 |
|  |  | (b) Indicate number of participants with missing data for each variable of interest | 9, Table 1 |
| Outcome data | 15* | Report numbers of outcome events or summary measures | 10-12, Table S3 |
| Main results | 16 | (*a*) Give unadjusted estimates and, if applicable, confounder-adjusted estimates and their precision (eg, 95% confidence interval). Make clear which confounders were adjusted for and why they were included | Table 3 |
|  |  | (*b*) Report category boundaries when continuous variables were categorized | p13-14 |
|  |  | (*c*) If relevant, consider translating estimates of relative risk into absolute risk for a meaningful time period | N/A |
| Other analyses | 17 | Report other analyses done—eg analyses of subgroups and interactions, and sensitivity analyses | N/A |
| Discussion | | | |
| Key results | 18 | Summarise key results with reference to study objectives | 14-16 |
| Limitations | 19 | Discuss limitations of the study, taking into account sources of potential bias or imprecision. Discuss both direction and magnitude of any potential bias | 17 |
| Interpretation | 20 | Give a cautious overall interpretation of results considering objectives, limitations, multiplicity of analyses, results from similar studies, and other relevant evidence | 14-16 |
| Generalisability | 21 | Discuss the generalisability (external validity) of the study results | 17 |
| Other information | | | |
| Funding | 22 | Give the source of funding and the role of the funders for the present study and, if applicable, for the original study on which the present article is based | Title page |

TABLE S2 **Questionnaire**

| **Section 1** | |
| --- | --- |
| **Question** | **Response option** |
| 1. Do you have enough time to look after your own diet? | Yes; No; I don’t know; Not applicable |
| 2. Do you follow a low-fat diet?  This response enabled categorising each responder as a current, past, or non-follower, past follower, or current follower of a low-fat diet.  Non-followers skipped the next three questions. | No; No, but I've tried following a low-fat diet in the past; Yes |
| 3. How long have you been following/did you follow a low-fat diet? | Less than four weeks; Less than three months; Less than six months; Less than 12 months; Over one year; Longer than five years |
| 4. Why do/did you follow a low-fat diet? | To help me to manage my BAD; To help me to manage my weight; To help me to look after my health; For another reason |
| 5. Which were useful sources of information for:  a) learning about a low-fat diet?  b) sticking to a low-fat diet? | GP; Gastroenterologist; Dietitian; Nutritionist; Other complementary or alternative practitioner; Family; Friends/colleagues; Books; Internet: applications; Internet searches, social media, blogs other; None |
| **Section 2** | |
| **Attitude:**  The extent to which they think that reducing fat intake is a good thing to do | |
| a. Having less fat in my diet could be beneficial to my health | |
| b. Having less fat in my diet is necessary for my health | |
| c. Having less fat in my diet would be pleasant for me | |
| d. Having less fat in my diet would be convenient for me | |
| e. My food costs would be less if I reduced how much fat is in my diet. This includes food and beverages from grocery shopping and eating away from home | |
| f. If I were to have less fat is in my diet I wouldn't need extra time to buy food or meals | |
| g. If I were to have less fat is in my diet I wouldn't need extra time to prepare or cook my meals | |
| **Subjective norm:**  The extent to which they agree that others important to them approve of them reducing fat intake | |
| h. People important to me, such as my family, friends and doctor, think that I should eat less fatty food | |
| i. People important to me, such as my family and friends, do not have lots of fatty or fried food in their usual diet | |
| **Perceived behavioural control:**  The extent to which they believe that reducing fat intake is up to them | |
| j. If I wanted to, it would be easy for me to have less fat in my diet | |
| k. If I wanted to, I would know what I to do to have less fat in my diet | |
| l. If I wanted to, I would have the self-discipline to have less fat in my diet | |
| m. Whether I have less fat in my diet or not is completely up to me | |
| **Perceived food-related symptom knowledge:**  The extent to which they believe that reducing fat intake improves their symptoms | |
| n. Having less fat in my diet might improve my diarrhoea | |
| o. Having less fat in my diet might improve my abdominal pain | |
| p. Having less fat in my diet might improve my bloating | |
| q. Having less fat in my diet might improve my wind | |
| r. Having less fat in my diet might help me to control my gut symptoms | |
| **Behavioural intention to reduce fat intake**:  The likelihood of reducing fat intake | |
| s. If I am offered the guidance, I want to cut back on how much fat is in my diet | |
| t. If I am offered the opportunity, I want to cut back on how much fat is in my diet | |

TABLE S3 **Agreement scores for the predictor variables on intention to reduce fat intake**

| **Predictor variable** | **Whole cohort** | | | **Low-fat diet following group** | | | | | |
| --- | --- | --- | --- | --- | --- | --- | --- | --- | --- |
|  |  |  |  | **Current follower** | **Past follower** | **Non- follower** | p-value after Bonferroni correction | | |
|  | n | Median  (IQR) | p-value | Median  (IQR) | Median  (IQR) | Median  (IQR) | CvP | CvN | PvN |
| **Attitude:** Having less fat in my diet … | | | | | | | | | |
| Could be beneficial to my health | 377 | 5.0  (4.0-6.0) | 0.35 | 5.0  (4.0 - 6.0) | 5.0  (4.0 - 6.0) | 5.0  (3.0 - 6.0) | 1.00 | 0.54 | 1.00 |
| Is necessary for my health | 392 | 5.0  (3.0-6.0) | **<0.01** | 5.0  (4.0- 6.0) | 5.00  (3.0 6.0) | 4.0  (2.0-6.0) | **0.04** | **0.01** | 1.00 |
| Would be pleasant for me | 393 | 3.0  (2.0-5.0) | 0.06 | 4.0  2.0-5.0 | 3.0  2.0 -5.0 | 3.0  1.0-4.0 | 0.41 | 0.08 | 0.92 |
| Would be convenient for me | 394 | 3.0  (1.0-4.0) | 0.69 | 3.0  1-5.0 | 3.0  1.0-4.0 | 2.0  1.0-4.0 | 1.00 | 1.00 | 1.00 |
| Food costs would be less | 370 | 2.0  (0-2.0) | 0.29 | 2.0  0-3.0 | 2.0  0-3.0 | 2.0  0-3.0 | 0.36 | 1.00 | 1.00 |
| Groceries: I would not need extra time | 383 | 2.0  (0-4.0) | 0.90 | 2.0  0-3.0 | 2.0  1.0-4.0 | 2.00  2.0-3.8 | 0.09 | 1.00 | 1.00 |
| Food prep: I would not need extra time | 380 | 1.0  (0-3.0) | 0.08 | 1.0  0-3.0 | 2.0  0-4.0 | 2.00  0-3.5 | 0.89 | 0.79 | 1.00 |
| **Subjective norm:** People important to me, such as:  h. my family, friends and doctor, think that I should eat less fatty food  i. my family and friends, do not have lots of fatty or fried food in their usual diet | | | | | | | | | |
| h. | 344 | 2.0  0-4.0 | 0.13 | 2.0  0-4.0 | 2.0  0-4.0 | 3.0  1.0-4.0 | 0.13 | 1.00 | 1.00 |
| i. | 368 | 3.0  1.0-4.8 | 0.17 | 3.0  1.0-5.0 | 3.0  1.0-4.0 | 3.0  1.0-4.0 | 0.36 | 0.40 | 1.00 |
| **PBC:** If I wanted to, it/I would … to have less fat in my diet | | | | | | | | | |
| Be easy | 394 | 3.0  2.0-5.0 | 0.95 | 3.0  2.0-5.0 | 3.0  2.0-5.0 | 3.0  2.0-5.0 | 1.00 | 1.00 | 1.00 |
| Know what I would have to do | 388 | 5.0  3.0-6.0 | **<0.01** | 5.0  4.0-6.0 | 4.0  2.0-5.0 | 4.0  2.8-6.0 | **<0.01** | 0.06 | 1.00 |
| Have the self-discipline | 386 | 4.0  2.0-5.3 | **<0.01** | 5.0  3.0-6.0 | 4.0  2.0-5.0 | 4.0  2.5-6.0 | **<0.01** | 0.92 | 0.30 |
| Be completely up to me… | 381 | 5.0  3.0-6.0 | 0.09 | 5.0  3.0-6.0 | 4.0  3.0-6.0 | 5.0  3.0-6.0 | 0.14 | 1.00 | 0.26 |
| **Perceived food-related symptom knowledge:** Having less fat in my diet might … | | | | | | | | | |
| Improve my diarrhoea | 388 | 5.0  (2.0-6.0) | **<0.01** | 5.0  3.0-6.0 | 4.0  4.0-5.0 | 4.0  3.0-5.0 | **<0.01** | 0.17 | 0.69 |
| Improve my abdominal pain | 369 | 4.0  (2.0-5.0) | **<0.01** | 5.0  2.0-6.0 | 3.0  2.0-5.0 | 4.0  3.0-5.0 | **<0.01** | 0.44 | 0.86 |
| Improve my bloating | 370 | 4.0  (2.0-5.0) | **0.03** | 4.0  2.0-5.0 | 3.0  2.0-4.0 | 4.0  2.0-5.0 | **0.03** | 1.00 | 0.56 |
| Improve my wind | 352 | 4.0  (2.0-5.0) | **0.02** | 4.0  2.0-5.0 | 3.0  2.0-4.0 | 4.0  2.0-5.0 | **0.02** | 1.00 | 0.32 |
| Help me to control my gut symptoms | 388 | 4.0  (2.0-5.0) | **0.02** | 4.0  2.0-6.0 | 3.0  2.0-5.0 | 4.0  3.0-5.0 | **0.02** | 1.00 | 0.66 |
| **Behavioural intention:** If offered the guidance/opportunity, I intend to cut back on how much fat is in my diet. | | | | | | | | | |
| **Guidance** | 370 | 5.0  3.0-6.0 | 0.39 | 5.0  3.0-6.0 | 4.0  3.0-6.0 | 4.0  3.0-6.0 | 0.74 | 0.84 | 1.00 |
| **Opportunity** | 375 | 5.0  3.0-6.0 | 0.80 | 5.0  3.0-6.0 | 4.0  3.0-5.0 | 5.0  2.0-5.0 | 1.00 | 1.00 | 1.00 |

Food prep, food preparation; CvP, Current followers versus Past followers; CvN, Current followers versus non-followers; PvN, Past followers versus non-followers
